# Selection of entropy-measure parameters for knowledge discovery in heart rate variability data

**DOI:** 10.1186/1471-2105-15-S6-S2

**Published:** 2014-05-16

**Authors:** Christopher C Mayer, Martin Bachler, Matthias Hörtenhuber, Christof Stocker, Andreas Holzinger, Siegfried Wassertheurer

**Affiliations:** 1 AIT Austrian Institute of Technology, Health & Environment Department, Biomedical Systems, Donau-City-Str. 1, 1220 Vienna, Austria; 2 Vienna University of Technology, Institute for Analysis and Scientific Computing, Wiedner Hauptstr. 8-10, 1040 Vienna, Austria; 3 Medical University Graz, Institute of Medical Informatics, Auenbruggerplatz 2/9/V, 8036 Graz, Austria

**Keywords:** entropy, parameter selection, comparison, knowledge discovery

## Abstract

**Background:**

Heart rate variability is the variation of the time interval between consecutive heartbeats. Entropy is a commonly used tool to describe the regularity of data sets. Entropy functions are defined using multiple parameters, the selection of which is controversial and depends on the intended purpose. This study describes the results of tests conducted to support parameter selection, towards the goal of enabling further biomarker discovery.

**Methods:**

This study deals with approximate, sample, fuzzy, and fuzzy measure entropies. All data were obtained from PhysioNet, a free-access, on-line archive of physiological signals, and represent various medical conditions. Five tests were defined and conducted to examine the influence of: varying the threshold value *r *(as multiples of the sample standard deviation *σ*, or the entropy-maximizing *r*_Chon_), the data length *N*, the weighting factors *n *for fuzzy and fuzzy measure entropies, and the thresholds *r_F _*and *r_L _*for fuzzy measure entropy. The results were tested for normality using Lilliefors' composite goodness-of-fit test. Consequently, the p-value was calculated with either a two sample t-test or a Wilcoxon rank sum test.

**Results:**

The first test shows a cross-over of entropy values with regard to a change of *r*. Thus, a clear statement that a higher entropy corresponds to a high irregularity is not possible, but is rather an indicator of differences in regularity. *N *should be at least 200 data points for *r *= 0.2 *σ *and should even exceed a length of 1000 for *r *= *r*_Chon_. The results for the weighting parameters *n *for the fuzzy membership function show different behavior when coupled with different *r *values, therefore the weighting parameters have been chosen independently for the different threshold values. The tests concerning *r_F _*and *r_L _*showed that there is no optimal choice, but *r *= *r_F _*= *r_L _*is reasonable with *r *= *r*_Chon _or *r *= 0.2*σ*.

**Conclusions:**

Some of the tests showed a dependency of the test significance on the data at hand. Nevertheless, as the medical conditions are unknown beforehand, compromises had to be made. Optimal parameter combinations are suggested for the methods considered. Yet, due to the high number of potential parameter combinations, further investigations of entropy for heart rate variability data will be necessary.

## Background

Heart rate variability (HRV) is the variation of the time interval between consecutive heartbeats. It highly depends on the extrinsic regulation of the heart rate (HR) and reflects the balance between the sympathetic and the parasympathetic nervous system [1]. Batchinsky et al. [2] develop a collection of methods for using HRV to describe regular periodic oscillations in the heart rate, attributed to the vagal and/or sympathetic branches of the autonomic nervous system.

Research on HRV has attracted considerable attention in the fields of psychology and behavioral medicine. It has its origin in the search for non-invasive correlates of injury severity which can be extracted from available signals in order to discover new cardiac biomarkers [2]. These signals are usually ones that are routinely measured, and include sources like a photoplethysmogram or the electrocardiogram (ECG) [3]. Electrocardiography is an interpretation of the electrical activity of the heart over some period of time. It is a non-invasive procedure, using electrodes attached to the surface of the skin, and is commonly used to measure the heart rate, the regularity of the beats, and characterize properties or injuries in the heart chamber. An *R-to-R interval (RRI) *describes the latency between two consecutive R peaks in the ECG. The RRI time series are used as input for the determination of HRV parameters.

In studies of HRV, both time- and frequency-domain measures are typically used by practitioners and researchers [1,4]. Additionally, further knowledge about the subject's status can be discovered by the evaluation of certain patterns and shifts in an "apparent ensemble amount of randomness" of a stochastic process [5]. This randomness, as well as the predictability of this process, can be measured by entropy [6]. Thus, it is a commonly used tool to describe the regularity of large biomedical data sets. The more regulatory inputs a system has, the higher its irregularity is, due to interference of those regulatory systems. This assumption is also true for many biomedical systems such as HRV [7]. Therefore, it is a reasonable hypothesis that a more regular heart rate variability is connected to a defect in a regulatory system. To measure this irregularity, Pincus et al. proposed approximate entropy (ApEn) [8]. Further types of entropy were developed based on this method to improve it [9]. Because of its ability to measure regularity, entropy is widely used as a diagnostic tool in medicine to derive and discover biomarkers in large biomedical data. Its applications range from sudden infant death syndrome [7], to complexity analysis of intracranial pressure dynamics during periods of severe intracranial hypertension [10], to quantification of amplitude variations in mechanomyographic signals [11], to analysis of short gait data sets [12], to automatic detection of normal, pre-ictal, and ictal conditions from recorded electroencephalography signals [13], to the postural sway in stroke patients [14].

The approach to quantify the structural complexity (or, inversely, regularity) of the HRV is called heart rate complexity (HRC) and utilizes methods derived from nonlinear dynamics. Note that complexity and variability are not necessarily the same [15]. A periodical signal, such as a sine wave, is variable but not complex. This property allows complexity measures to ignore the complicated periodic oscillations to at least some extent [16].

To date, numerous entropy types are widely accepted as measures of the HRC. However, since their function is controlled by three, four or six parameters, there are many possible combinations to choose from. The selection of criteria for these parameters is controversial and heavily depends on the intended purpose and the data at hand [17]. The variation of only one parameter often results in highly non-linear behaviour [12]. Therefore, results of calculations with different parameters cannot be easily extrapolated from existing data, but have to be computed individually. Hence, the variation of several parameters in order to optimize the reliability of the results is a very time consuming process. To be used in daily routine, reasonable parameters have to be selected prior to the evaluation of HRC.

The determination of appropriate parameters for entropy in general [11,12,18-20] and for HRV applications in particular [16,17,21,22] is the subject of on- going research. A summary of the current knowledge regarding parameter selection is given in the subsection "Parameter selection". In order to verify the results of previous publications and to extend them by the usage of more entropy measures, this work focuses on the parameter selection for approximate, sample, fuzzy and fuzzy measure entropy and their implications for HRV data. The objective of this paper is to provide a reference for choosing parameters for HRV applications based on their influence on the different entropies' ability to distinguish significantly between pathological and non-pathological recordings. The main research questions addressed in this paper are: (1) what does the choice of the threshold value(s) mean for the entropies to be a direct measure of regularity, (2) how does the data length influence significance for different data sets, (3) how should the weighting factor(s) be chosen for fuzzy and fuzzy measure entropy, and finally, (4) what are the constraints for choosing the threshold values?

## Methods

In the following sections approximate (ApEn), sample (SampEn), fuzzy (FuzzyEn) and fuzzy measure entropy (FuzzyMEn) are described in detail. They are built on each other and ordered by increasing complexity. The number of parameters increases from three for ApEn to six for FuzzyMEn, with the basic parameters staying the same and being extended by new ones in each step. Afterwards, the challenge of parameter selection and the tests conducted for the parameters to the different entropy types are described. Finally, the data used for all performed tests are briefly described.

### Approximate entropy

The main idea behind approximate entropy is that a sequence is regular if a subsequence and an expansion of the subsequence are similar. It was developed by Pincus [8] and is calculated the following way.

Given a sample sequence {*u*_1_, . . . , *u_N _}*, a template length *m *and a threshold value *r*, the sequence is first split into overlapping sequences $\left\{X_{1}^{m},\ldots \;,X_{N-m+1}^{m}\right\}$ of length *m*, with $X_{i}^{m}:=\left\{u_{i},\ldots \;,u_{i+m-1}\right\}$. For example, for the input sequence {1, 2, 3, 4, 5} the overlapping sequences are {{1, 2, 3}, {2, 3, 4}, {3, 4, 5}} for *m *= 3. Next, define $C_{i}^{m}$ as the number of *j *= 1, . . . , *N *− *m *+ 1, for which $d\left(X_{i}^{m},X_{j}^{m}\right)<r$, where $d\left(X_{i}^{m},X_{j}^{m}\right)$ is the Chebyshev distance, i.e., the maximum distance between two elements of $X_{i}^{m}$and $X_{j}^{m}$. Constructing the same sequences, but with template length *m *+ 1, yields $C_{i}^{m+1}$. Then, $\phi^{m}$ and $\phi^{m+1}$ are defined as:

(1) $$\phi^{m}:=\frac{1}{N-m+1}\overset{N-m+1}{\underset{i=1}{\sum }}\text{ln}\left(\frac{C_{i}^{m}}{N-m+1}\right),\;\text{and}$$

(2) $${\phi^{m}}^{+1}:=\frac{1}{N-m}\overset{N-m}{\underset{i=1}{\sum }}\text{ln}\left(\frac{C_{i}^{m+1}}{N-m}\right).$$

The approximate entropy is then defined as $\text{ApEn }\left(m,r\right):=\underset{N\to \infty }{\text{lim}}\left(\phi^{m}-\phi^{m+1}\right)$, which can be estimated by $\text{ApEn }\left(m,r,N\right):=\phi^{m}-\phi^{m+1}$.

### Sample entropy

Richman and Moorman showed in [23] that approximate entropy is biased towards regularity. Thus, they modified it to sample entropy. The main difference between the two is that sample entropy does not count self-matches, and only compares the first *N − m *subsequences instead of all *N − m *+ 1, so the same amount of subsequences are used in *ϕ^m ^*and *ϕ*^*m*+1 ^[23]. It is calculated in the following way.

Given a sample sequence {*u*_1_*, . . . , u_N_*}, a template length *m*, and a threshold value *r*, first the overlapping sequences, $\left\{X_{1}^{m},\ldots \;,X_{N-m}^{m}\right\}$ are constructed as for ApEn. As opposed to ApEn, $C_{i}^{m}$ is now defined as the number of *j *= 1, . . . , *N − m*, for which $d\left(X_{i}^{m},X_{j}^{m}\right)<r$ where *i *≠ *j*, where $d\left(X_{i}^{m},X_{j}^{m}\right)$ is again the Chebyshev distance. Applying the same for template length *m *+ 1 results in:

(3) $$\phi^{m}:=\frac{1}{N-m}\overset{N-m}{\underset{i=1}{\sum }}\frac{C_{i}^{m}}{N-m-1},\;\;\text{and}$$

(4) $$\phi^{m+1}:=\frac{1}{N-m}\overset{N-m}{\underset{i=1}{\sum }}\frac{C_{i}^{m+1}}{N-m-1}.$$

Sample entropy is then defined as $\text{SampEn}\left(m,r\right):=\underset{N\to \infty }{\text{lim}}\left(\text{ln}\phi^{m}-\text{ln}\phi^{m+1}\right),$ which can be estimated by $\text{SampEn}\left(m,r,N\right):=\text{ln}\phi^{m}-\text{ln}\phi^{m+1}.$

### Fuzzy entropy

**Figure 1:**
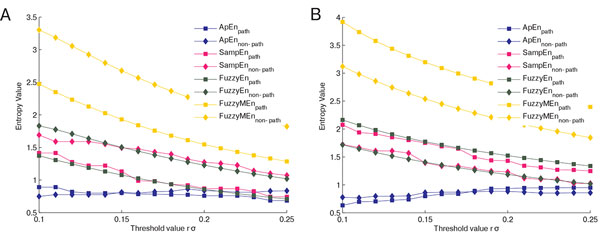
**Entropy values for different threshold values (Flip-flop effect)**. Entropy values for different threshold values *r *= *r_L _*= *r_F _*∈ [0.1σ, 0.25σ] for pathological (square markers) and non-pathological (diamond markers) data sets, parameters: *m *= 2, *n *= *n_L _*= 3, *n_F _*= 2, databases: chf2 vs. np (A) and mit vs. np (B)

ApEn and SampEn are very sensitive with respect to the threshold parameter *r*. They show, on one hand, a very abrupt behavior, and on the other hand less significance for small *r*, as was discussed in [9] and can be seen in figure 1. To soften these effects, Chen et al. developed fuzzy entropy, which uses a fuzzy membership function instead of the Heaviside function [9]. FuzzyEn is calculated as follows.

Let *m *and *r *again be the template length and the threshold value and *n *a weight for the fuzzy membership function. Sequences $\left\{X_{1}^{m},\ldots \;,X_{N-m+1}^{m}\right\}$ are defined as for ApEn and SampEn from an input sequence {*u*_1_, . . . , *u*_N_}, with $X_{i}^{m}:=\left\{u_{i},\ldots \;,u_{i+m-1}\right\}.$ Next, these sequences are transformed into sequences, $\left\{{\bar{X}}_{1}^{m},\ldots \;,{\bar{X}}_{N-m+1}^{m}\right\}$, where ${\bar{X}}_{i}^{m}:=\left\{ui-u0_{i}\overset{\;}{,}\ldots \;,{\;u}_{i+m-1}-u0_{i}\right\}$ and $u0_{i}$ is the mean value of $X_{i}^{m}$, i.e.,

(5) $$u0_{i}:=\overset{m-1}{\underset{j=0}{\sum }}\frac{u_{i+j}}{m}.$$

Next, using a fuzzy membership function *x *→ *µ*(*x, n, r*) a membership matrix *D^m ^*is calculated, where each element is defined as $D_{i,j}^{m}:=\mu \left(d\left({\bar{X}}_{i}^{m},{\bar{X}}_{j}^{m}\right),n,r\right)$. According to Chen et al. [9], fuzzy membership functions must be continuous and convex. The first property guarantees only slow similarity changes, and by the second condition self-similarity is a maximum of the function. For all tests conducted in this study, $x\to e^{-{\left(x/r\right)}^{n}}$ was chosen as the fuzzy membership function as in [9]. For *n → ∞ *this fuzzy membership function converges to the Heaviside function. These steps are repeated for template length *m *+ 1 to get *D^m+1^*. Consequently, $\phi^{m}$ and $\phi^{m+1}$ are calculated the following way:

(6) $$\phi^{m}:=\frac{1}{N-m}\overset{N-m}{\underset{i=1}{\sum }}\overset{N-m}{\underset{j=1,j\neq i}{\sum }}\frac{D_{i,j}^{m}}{N-m-1},\;\;\text{and}$$

(7) $$\phi^{m+1}:=\frac{1}{N-m}\overset{N-m}{\underset{i=1}{\sum }}\overset{N-m}{\underset{j=1,j\neq i}{\sum }}\frac{D_{i,j}^{m+1}}{N-m-1}.$$

Fuzzy entropy is defined as $\text{FuzzyEn}\left(m,r,n\right):=\underset{N\to \infty }{\text{lim}}\left(\text{ln}\phi^{m}-\text{ln}\phi^{m+1}\right)$ and can be estimated by $\text{FuzzyEn}\left(m,r,n,N\right):=\text{ln}\phi^{m}-\text{ln}\phi^{m+1}.$

### Fuzzy measure entropy

Liu et al. proposed adding a function for global similarity to the fuzzy entropy and called the combination fuzzy measure entropy [24]. It can be calculated as follows.

Given a data sequence {*u*_1_*, . . . , u_N_*}, a template length *m*, two threshold values *r_L _*and *r_F_*, and two weighting parameters *n_L _*and *n_F_* (*r_L_* and *n_L_* correspond to the local term and *r_F_* and *n_F_* to the global one), a sequence $\left\{X_{1}^{m},\ldots \;,X_{N-m+1}^{m}\right\}$ is constructed like before. In the next step, it is transformed into a local sequence $\left\{XL_{1}^{m},\ldots \;,XL_{N-m+1}^{m}\right\}$ and a global sequence, $\left\{XF_{1}^{m},\ldots \;XF_{N-m+1}^{m}\right\}$, with $XL_{i}^{m}:=X_{i}^{m}-u0_{i}$ where $u0_{i}$ is defined as in (5) and $XF_{i}^{m}:=X_{i}^{m}-u_{\text{mean}},$ and $u_{\text{mean}}$is the mean value of the complete data sequence {*u*_1_, . . . , *u_N_*}. Using the local and the global parameters for the fuzzy membership functions, the matrices *DL^m ^*and *DF^M ^*are defined as $DL_{i,j}^{m}:=\mu \left(d\left(XL_{i}^{m},XL_{j}^{m}\right),n_{L},r_{L}\right)$ and $DF_{i,j}^{m}:=\mu \left(d\left(XF_{i}^{m},XF_{j}^{m}\right),n_{F},r_{F}\right)$. Afterwards, all these steps are repeated for template length *m *+ 1 to get *DL*^*m*+1^ and *DF*^*m*+1^. Finally, $\phi_{L}^{m},\phi_{F}^{m},\phi_{L}^{m+1}$ and $\phi_{F}^{m+1}$ are defined as:

(8) $$\phi_{L}^{m}:=\frac{1}{N-m}\overset{N-m}{\underset{i=1}{\sum }}\overset{N-m}{\underset{j=1,j\neq i}{\sum }}\frac{DL_{i,j}^{m}}{N-m-1},$$

(9) $$\phi_{F}^{m}:\frac{1}{N-m}\overset{N-m}{\underset{i=1}{\sum }}\overset{N-m}{\underset{j=1,j\neq i}{\sum }}\frac{DF_{i,j}^{m}}{N-m-1},$$

(10) $$\phi_{L}^{m+1}:=\frac{1}{N-m}\overset{N-m}{\underset{i=1}{\sum }}\overset{N-m}{\underset{j=1,j\neq i}{\sum }}\frac{DL_{i,j}^{m+1}}{N-m-1},\;\;\text{and}$$

(11) $$\phi_{F}^{m+1}:=\frac{1}{N-m}\overset{N-m}{\underset{i=1}{\sum }}\overset{N-m}{\underset{j=1,j\neq i}{\sum }}\frac{DF_{i,j}^{m+1}}{N-m-1}.$$

Fuzzy measure entropy is then defined as FuzzyMEn(*m, r_L_, r_F_, n_L_, n_F_*) := $\underset{N\to \infty }{\text{lim}}\left(\text{ln}\phi_{L}^{m}-\text{ln}\phi_{L}^{m+1}+\text{ln}\phi_{F}^{m}-\text{ln}\phi_{F}^{m+1}\right)$, which can be estimated by $\text{FuzzyMEn}\left(m,\;r_{L},\;r_{F},\;n_{L},\;n_{F},\;N\right)\;:=\left(\text{ln}\phi_{L}^{m}-\text{ln}\phi_{L}^{m+1}+\text{ln}\phi_{F}^{m}-\text{ln}\phi_{F}^{m+1}\right)$.

### Parameter selection

As one can see in the description of each entropy, the various entropy types have three, four or six parameters. Thus, there are many possible combinations to choose from. The parameters which are varied in the test cases, their ranges and values mentioned in the literature, and the choice of certain fixed parameters are described here.

The most common choice for the template length is *m *= 2, as it was recommended by Pincus and Goldberger for ApEn [7], by Yentes et. al for SampEn [12], and confirmed by other studies, e.g. [20]. A false nearest neighbor method is also sometimes used, but according to Chon et al. the standard choice leads to the statistically best solutions for ApEn for human heart rate variability data [25]. In [9,24], the template length is set to *m *= 2 for other entropy types as well. For comparability, this value was used for all tests.

Some publications describe a sensitivity of the entropies to the data length *N *[1,6,8]. Therefore, the significance of the entropies calculated of parts of the heart rate variability data has been tested with increasing data set size.

For the threshold parameter *r*, Pincus suggested in [8] to choose a value between 0.1 *σ *and 0.25 *σ*, where *σ *is the sample standard deviation of the data sequence. This is also the standard range in most publications, with the most common choice of *r *= 0.2 *σ *[17,21,23,24,26]. To examine threshold parameters in the standard range, they were tested with heart rate variability data.

In [19,27], the so called flip-flop effect is described, where for some values of the threshold value *r *a signal has a higher entropy compared to another and for other choices of *r *a lower one. This is also shown in [21] for heart rate variability data. To test for this effect, various entropies of signals from one database were calculated with parameters inside the standard range. Lu et al. also showed this effect in [26]. Therefore, they proposed choosing *r *∈ [0.1 *· σ*, 1.0 · *σ*] for ApEn in such a way as to maximize the entropy value. Since finding a maximum is computationally very expensive, Chon et al. created in [25] an empirical formula to calculate an *r*, hereinafter called *r*_Chon_, which approximates a maximizing *r*. For *m *= 2, it can be formulated as:

(12) $$r_{\text{Chon}}:=\left(-0.036+0.26\sqrt{\sigma_{1}\text{/}\sigma_{2}}\right)/\sqrt[{4}]{N\text{/}1000},$$

where *σ*_1 _is the standard deviation of the distances in the data sequence, i.e., the standard deviation of {(*u*_1 _− *u*_2_), . . . , (*u*_*N*−1 _− *u_N_*)}, and *σ*_2_ the standard deviation of the complete data sequence. This formula was derived from non-physiological data and Liu et al. showed in [17] that it is not always a good approximation of the maximizing *r*, but actually leads to more significant results than the maximizing *r*, when applied to heart rate variability data.

Regarding fuzzy function parameters, Chen et al. [9] used *n *= 2 and *r *= 0.2 *σ *for test signals. They described in [9] that for a larger *n*, the closer data points are weighted more strongly. Liu et al. [24] used the weighting factors *n_L _*= 3, *n_F _*= 2 and *r_L _*= *r_F _*= 0.2*σ *for heart rate variability analysis. Their choices for *n_L _*and *n_F _*were given without any motivation.

### Test cases

To get further knowledge of the parameters, heart rate variability data were used to compare different choices of *r_L_, r_F _*and *n, n_L _*and *n_F _*with respect to the resulting significance of the statistical tests comparing pathological and normal cases.

The following test cases using the data described in the Data section have been conducted to answer the research questions stated in the Background section and to support an "optimal" parameter selection for all entropy types for heart rate variability data. For each research question, one test case has been designed (except for the third, which is covered in two test cases: one for each entropy type under consideration). All tests have been conducted consecutively. Fixed parameters have been taken from literature or based on the outcomes of preceding tests.

#### Test case 1

Variation of the threshold values *r *= *r_L _*= *r_F _*within the standard interval [0.1 *σ*, 0.25 *σ*] [8] to show its influence on the entropy values and the aforementioned flip-flop effect [21,27]. Fixed parameters were *m *= 2, *n *= *n_L _*= 3 and *n_F _*= 2 [7,24]. For data length *N*, the data length of the shortest RR interval sequence of the available data *N *= 1126 sets was used.

#### Test case 2

Variation of the data length *N *= *x *· 110 with *x *∈ [1, 10] to show its influence on the significance. Maximum *N *was 1100 due to the length of the available test data. Fixed parameters were *m *= 2, *n *= *n_L _*= 3, *n_F _*= 2 and *r *= *r*_Chon _or *r *= 0.2 *σ *[7,17,21,23-26].

#### Test case 3

Variation of the weighting factor *n *for FuzzyEn in the interval [1, 6] to show its influence on the significance. Fixed parameters were *m *= 2 and *r *= *r*_Chon _or *r *= 0.2 *σ *[7,17,21,23-26]. *N *= 1000 was chosen based on the results of test case 2.

#### Test case 4

Variation of the weighting factors *n_L_* and *n_F_* for FuzzyMEn in the interval [1, 6] to show their influence on the significance. Fixed parameters were *m *= 2 and *r_L _*= *r_F _*= *r*_Chon _or *r_L _*= *r_F _*= 0.2 *σ *[7,17,21,23-26]. *N *= 1000 was chosen based on the results of test case 2.

#### Test case 5

Variation of the threshold values *r_L _*and *r_F _*for FuzzyMEn in an interval of [0.25 *· r*_Chon_, 6 *· r*_Chon_] and in an interval of [0.1 *σ*, 0.25 *σ*][8] to show their influence on the significance. The parameter *m *= 2 was fixed [7]. *N *= 1000, *n_L _*and *n_F _*were chosen based on the results of the previous tests.

In order to test for their statistical significance, the calculated entropies were first tested for normality using Lilliefors' composite goodness-of-fit test [28]. If this test was positive for all results within a subset of a test case (i.e., certain database and/or choice for *r*), the p-value was calculated with a two sample t-test, and otherwise a Wilcoxon rank sum test was performed. To ensure comparability, the same statistical test was used for each subset of a test case.

Since statistical tests used in this work are based on the same null hypothesis, the same subject groups, the same endpoints and only slight variations of the analysis method, interaction of the observed results is not only possible, but highly probable. On the other hand, p-value adjustments such as the commonly used Bonferroni correction assume uncorrelated endpoints and are therefore considered inappropriate for the tasks in this work [29]. Besides, the aim of this work is not to test whether there is a difference between groups, but to investigate the ability of varied methods to detect those differences.

Due to the high computational complexity of the tests conducted (e.g., the calculation of the variation of the weighting factors *n_L _*and *n_F _*for FuzzyMEn takes several hours for only one *r *and one comparison of databases), the authors had to refrain from more robust randomized testing strategies (i.e., the permutation of the original data), since the computation time would multiply by at least a thousand times.

### Data

All data used for the described tests have been taken from http://Physionet.org[30], a free-access, on-line archive of physiological signals. They are described in detail in this section.

To create a control group all databases described as non-pathological were combined into one database, afterwards called *np*. This database contains the Normal Sinus Rhythm RR Interval Database, which consists of the beat annotations of 54 long-term ECG recordings, digitized at 128 samples per second, of subjects with normal sinus rhythm (30 men, aged 28.5 to 76, and 24 women, aged 58 to 73). Furthermore, it includes the MIT-BIH Normal Sinus Rhythm Database, which consists of 18 long-term recordings (5 men, aged 26 to 45, and 13 women, aged 20 to 50) digitized at 128 samples per second. The Fantasia Database of 120-minute recordings of twenty young (10 men and 10 women; 21 - 34 years old) and twenty elderly (10 men and 10 women; 68 - 85 years old) healthy subjects with ECG digitized at 250 Hz was also used [31]. The record "fantasia/f1o09" had to be excluded due to its high number of supraventricular premature beats. This results in a total database size of 111 recordings. RR intervals greater than 2.5 seconds were excluded to ignore artifacts.

Two databases were used to search for pathological effects. One was the Congestive Heart Failure RR Interval Database, afterwards referred to as *chf2*, which includes 29 long-term ECG recordings, with a sampling frequency of 128 Hz, of subjects aged 34 to 79 (8 men and 2 women; gender not known for the remaining subjects) with congestive heart failure (NYHA classes I, II, and III) [32].

The second one was the MIT-BIH Arrhythmia Database, afterwards called *mit*, which contains 48 half-hour recordings, sampled with a frequency of 360 Hz, from 47 subjects (25 men aged 32 to 89 years and 22 women aged 23 to 89 years) [33]. It contains a set of randomly chosen signals and 25 signals especially chosen to include examples of uncommon but clinically important arrhythmias recorded at the BIH Arrhythmia Laboratory [33].

The two databases *chf2 *and *mit *were always evaluated separately to keep the results as homogeneous as possible and to avoid the mutual neutralization of ab- normalities. To ensure comparability, the data length *N *was equal for all recordings. Longer recordings were cropped at the beginning and the end in equal shares.

In a way, this work can be considered as a pilot study, as only previously recorded data are used. Furthermore, the findings of this work will be incorporated in follow-up studies.

## Results

The following sections show the results of our tests, which are described in the Test cases section above. An overview of these results is given in Table 1.

**Table 1:** Summary of the best results achieved for each test case.


**Test case number**	**Test case description**	**Results with**	
		***r *= *r*_Chon_**	***r *= 0.2 σ**

1	Variation of the threshold values *r *= *r_L _= r_F _*within the standard interval [0.1 σ, 0.25 σ]	Not applicable	Flip-flop effect verified for ApEn but not for other entropies
2	Variation of the data length *N *= *x *· 110 with *x *∈ [1,10]	Significant with *N *> 1000	Significant with *N *> 200
3	Variation of the weighting factor *n *for FuzzyEn in the interval [1,6]	Significant with *n *< 3	Significant with *n *= 1
4	Variation of the weighting factors *n_L _*and *n_F _*for FuzzyMEn in the interval [1,6]	Best results with *n_L _*= 2, *n_F _*= 1	Best results with *n_L _*= 1, *n_F _*= 3
5	Variation of *r_L _*and *r_F _*for FuzzyMEn in an interval of [0.25 · *r*_Chon_, 6 · *r*_Chon_] and in an interval of [0.1σ, 0.25σ]	inconclusive	

**Figure 2:**
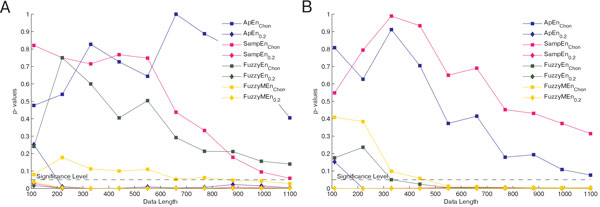
**Influence of data length on significance**. The influence of the data length on the significance of the entropy types, parameters: *m *= 2, *r *= *r_L _*= *r_F _*= *r*_Chon _or *r *= *r_L _*= *r_F _*= 0.2σ, *n *= *n_L _*= 3, *n_F _*= 2, databases: chf2 vs. np (A) and mit vs. np (B)

**Figure 3:**
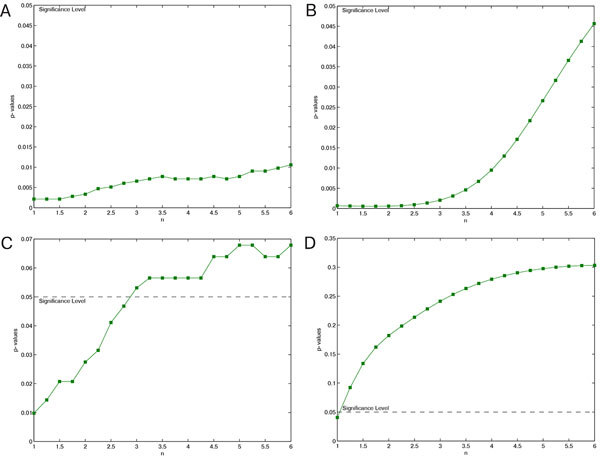
**Significance of FuzzyEn for different choices of *n***. Significance of FuzzyEn for different choices of n for *r *= *r*_Chon _(A, C) and *r *= 0.2σ (B, D); parameters: *m *= 2, *N *= 1000, databases: chf2 vs. np (A, B) and mit vs. np (C, D)

**Figure 4:**
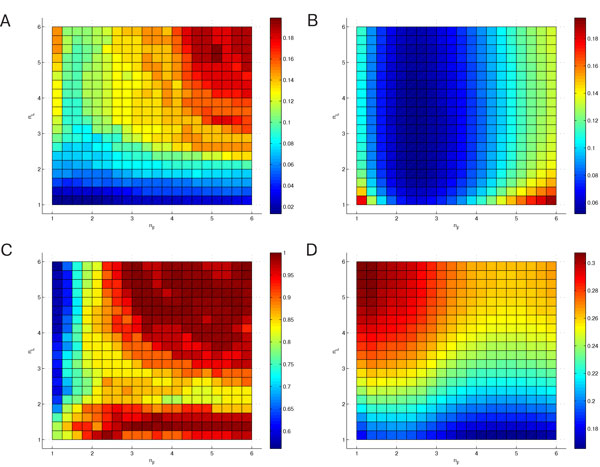
**Significance of FuzzyMEn for different choices of *n_L _*and *n_F_***. Significance of FuzzyMEn for different choices of *n_L _*and *n_F _*for *r_L _*= *r_F _*= *r_Chon _*(A, C) and *r_L _*= *r_F _*= 0.2 σ (B, D); parameters: *m *= 2, *N *= 1000, databases: chf2 vs. np (A, B) and mit vs. np (C, D)

**Figure 5:**
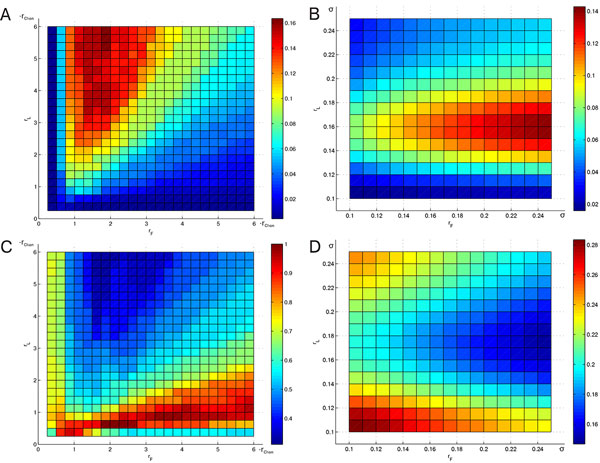
**Significance of FuzzyMEn for different choices of *r_L _*and *r_F_***. Significance of FuzzyMEn for different choices of *r_L _*and *r_F _*in an interval of [0.25 · *r*_Chon_; 6 · *r*_Chon_] with *n_L _*= 2, *n_F _*= 1 (A, C) and in an interval of [0.1σ, 0.25σ] with *n_L _*= 1, *n_F _*= 3 (B, D); parameters: *m *= 2, *N *= 1000, databases: chf2 vs. np (A, B) and mit vs. np (C, D)

The results were tested for normality using Lilliefors' composite goodness-of-fit test. Throughout the whole test case 2, which combines results of two different ways to determine *r * (see figure 2), data were either normally or not normally distributed. Hence, entropy values were compared using the Wilcoxon rank sum test. Entropies calculated with *r *as multiple of *σ *were distributed normally in our test scenarios and could therefore be compared using the two sample t-test (as in figures 3 (B, D), 4 (B, D) and 5 (B, D)). Entropy values derived using *r *as multiple of *r*_Chon_, on the other hand, were not normally distributed and hence compared using Wilcoxon's rank sum test (as in figures 3 (A, C), 4 (A, C) and 5 (A, C)). Only findings of the same statistical test are combined in the following sections.

Figure 1 corresponds to test case 1 and shows the effect of different threshold values on the entropy values. One of the defining characteristics of FuzzyEn and FuzzyMEn, their relative insensitivity to changes in *r*, is clearly visible. The flip-flop effect can be observed for ApEn. For the *chf2 *database, entropy values are higher for pathological data where *r <*0.15 *σ*, whereas they are lower for *r >*0.15 *σ*. This behavior is reversed for the *mit *database, with lower entropy values for pathological data for *r <*0.18 *σ *and higher entropy values for pathological data for *r >*0.18 *σ*.

The results for test case 2, analyzing the sensitivity of the entropy types to the data length are represented in figure 2. The more data points evaluated, the higher the separation between pathological and non-pathological data. When using the threshold value *r *= 0.2 *σ*, significance is already reached with *N *≥ 200 data points, whereas with *r *= *r*_Chon _more data (*N *≥ 1000) are needed before significance is reached. Comparing the different methods when using *r *= 0.2 *σ*, one can see that they only differ when *N *is very small. With higher *N *, their behavior converges.

Figure 3 presents the results of test case 3, showing that the results become insignificant for *n >*3 with *r *= *r*_Chon_, and for *n >*1 with *r *= 0.2 *σ *in the *mit *database (C, D). The increasing p-values are below the significance level (*p <*0.05) for the *chf2 *database within the observed range of *n *(A, B).

Figure 4, corresponding to test case 4, shows a higher significance for *n_L _*≤ 2 and all values of *n_F_* (A), or in the case of *r *= 0.2 *σ *for *n_F_* ∈ [2, 3.5] (B). In figure 4 (C and D) the situation is different: the best results are achieved with *n_F _*< 1.5 and *n_L _*≥ 2 for *r *= *r*_Chon _in case C, whereas for *r *= 0.2 *σ *(D) the best performance is reached with *n_L _*< 1.5 and any *n_F_*.

The results for test case 5 are shown in figure 5. They represent the ability of FuzzyMEn to differentiate between *chf2 *and *np *(A and B) and *mit *and *np *(C and D) for different choices of the threshold values *r_F _*and *r_L_*, when chosen as multiples of *r*_Chon _in the range of [0.25 *· r*_Chon_, 6 *· r*_Chon_] (A, C), and calculated according to the results of test case 4 with *n_L _*= 2 and *n_F _*= 1, or inside the standard range of [0.1 *σ*, 0.25 *σ*] (B, D), calculated with *n_L _*= 1 and *n_F _*= 3. In (A), the best results are achieved with $r_{L}≲1\cdot r_{\text{Chon}}$ or $r_{F}≲1\cdot r_{\text{Chon}}$, whereas they have to be greater than or equal to 1 *· r*_Chon _in (C) for good performance. In (B), *r_L _<*0.12 *σ *or $r_{L}≳0.2\sigma$ yield significant results, however *rF *does not matter that much. In contrast, the best performance in (D) is achieved with $r_{F}≳0.18\sigma$ and *r_L_*∈ [0.14 *σ*, 0.22 *σ*].

## Discussion

As a summary of the following discussion of the previously presented results, the combinations of parameters which yielded the best results are listed in Table 2.

**Table 2:** Recommendations for different combinations of parameters.

*r *= *r_L _*= *r_F_*	*m*	*N*	*n *= *n_L_*	*n_F_*
*r*_Chon_	2	*>*1000	2	1
0.2 *σ*	2	*>*200	1	3

Some of the tests concerning the parameter selection showed no clear results. A couple of evaluations revealed contradicting results depending on the database (*chf2 *vs. *mit *) or on the chosen parameters (e.g., *r *= *r_L _*= *r_F _*= *r*_Chon _vs. *r *= *r_L _*= *r_F _*= 0.2*σ*). The latter is easy to overcome by choosing a different set of parameters based on the choice of the threshold value *r*. However, a compromise must be found in order to find parameters suitable for both databases.

One of the hardest difficulties lies in the choice of the threshold value *r *due to the flip-flop effect, i.e., for some parameters one data set has a higher entropy compared to another, but this order is reversed for different parameter choices [17,27]. This can occur for simple signals, but also when analyzing heart rate variability data, as in figure 1. This leads to difficulties with the interpretation of the entropy, i.e., the direct assignment of entropy values to pathological or non-pathological data without a given *r*. In our tests, the effect occurred for ApEn, but it is also reported to occur for SampEn and FuzzyEn as well by Boskovic et al. [27]. The flip-flop effect does not allow us to make a clear statement, as in [7-9,17], that a higher entropy corresponds to a higher irregularity. Since this effect can happen for all different entropy values [27], they should not be seen as a direct measure of regularity, but rather as an indicator of differences in regularity with regard to certain time periods.

Compared to the threshold value, the data length *N *seems to have a smaller effect on the ability of the entropy measures to differentiate pathological from non-pathological data sets. Generally, a larger *N *leads to a higher probability of significance. If possible, the data length *N *should be longer than 200 data points when using *r *= 0.2 *σ*. This finding is consistent with previous studies, e.g. [12]. Surprisingly, it should even exceed a length of 1000 data points when using *r *= *r*_Chon_, assuming a continuing trend in figure 2. Due to the length of the available test data, the range had to be restricted for this test. In case of HRV data, *N *is the number of recorded heartbeats and therefore proportional to the duration of the recording. Thus, to increase *N*, longer measurements are necessary. The Task Force of The European Society of Cardiology and The North American Society of Pacing and Electrophysiology [4] recommends a duration of five minutes for short time recordings, which would result in 300 data points at an average heart rate of 60 beats per second. This would be sufficient when using *r *= 0.2 *σ*, but not for *r *= *r*_Chon_. These considerations should be kept in mind when dealing with HRV data.

Due to the different behavior when varying *n, n_L _*and *n_F _*given different threshold parameters *r *= *r_L _*= *r_F _* = *r*_Chon _and *r *= *r_L _*= *r_F _* = 0.2 *σ*, the parameters *n, n_F _*and *n_L _*have been chosen independently for the different threshold values. This is no constraint to the method, since the choice of *r *is known beforehand anyway (and is not based on the potentially unknown medical condition of the subject). The values *n_L _*= 2 and *n_F _*= 1 for *r_L _*= *r_F _*= *r*_Chon_, and *n_L _*= 1 and *n_F _*= 3 for *r_L _*= *r_F _*= 0.2 *σ *showed better results than *n_L_*= 3, *n_F_* = 2 as proposed by Liu et al. in [24]. Unsurprisingly, similar values were found for *n*, as *n *for FuzzyEn equals *n_L _*for FuzzyMEn. For consistency, *n *= *n_L _*= 2 for *r *= *r*_Chon _and *n *= *n_L _*= 1 for *r *= 0.2 *σ *are recommended.

The tests concerning *r_F _*and *r_L _*showed that there is no optimal choice, since the results in figure 5 (A) and (C) as well as (B) and (D) contradict each other. Nevertheless, both *r_F _*= *r_L _*= *r*_Chon _and *r_F _*= *r_L _*= 0.2 *σ*, as described in the literature [9,24,25], are the most reasonable compromise between figure 5 (A) and (C), and (B) and (D), respectively.

Finally, a number of important limitations of this study need to be considered. First, this study is limited to previously recorded signals of only two different cardiac diseases due to the availability of data. As already mentioned in the Data section, a separate evaluation is warranted, to avoid the mutual neutralization of abnormalities. Furthermore, the template length was fixed to *m *= 2 for all calculations, as the number of possible variations of parameters would get too high otherwise and *m *= 2 seems to be a reasonable choice in all investigated literature, e.g., [8]. In [34] it was reported that spikes due to recording errors in heart rate variability data can disturb ApEn and SampEn. No tests were done to examine this behavior for the included entropies. Finally, the study did not evaluate the dependency of the entropies on age and gender as reported in literature [35,36].

## Conclusions

The results of the presented study clearly stress the need for further investigations of signal entropy for heart rate variability data. Given the wide range of different medical conditions of subjects, the assortment of available methods to calculate entropy, and their customizability with up to six degrees of freedom, it is almost impossible to cover all combinations in a single study. Future work will therefore be focused on overcoming the limitations of the presented work, i.e., extending the evaluations to other cardiac diseases, the variation of the template length *m*, investigating the robustness with respect to recording errors, and the relation of the parameter choice to age and gender.

## Competing interests

The authors declare that they have no competing interests.

## Authors' contributions

CM and MB have equally contributed to this work. CM participated in the design and coordination of the study, the interpretation of the results and drafted the manuscript. MB carried out the calculations and interpretation of the results and participated in drafting the manuscript. MH carried out the implementation of methods, calculations and interpretations. CS participated in the implementation. AH helped to draft the manuscript. SW participated in the design of the study and drafting the manuscript. All authors read and approved the final manuscript.

## Declarations

This research received no grant from any funding agency. Publication for this article has been funded by the research group.

This article has been published as part of *BMC Bioinformatics *Volume 15 Supplement 6, 2014: Knowledge Discovery and Interactive Data Mining in Bioinformatics. The full contents of the supplement are available online at http://www.biomedcentral.com/bmcbioinformatics/supplements/15/S6.
